# In Vitro Infection Dynamics of Japanese Encephalitis Virus in Established Porcine Cell Lines

**DOI:** 10.3390/pathogens10111468

**Published:** 2021-11-12

**Authors:** Shakirat A. Adetunji, Dmitriy Smolensky, Dana N. Mitzel, Jeana L. Owens, Carol G. Chitko-McKown, Natalia Cernicchiaro, Leela E. Noronha

**Affiliations:** 1 Center for Outcomes Research and Epidemiology, Department of Diagnostic Medicine/Pathobiology, College of Veterinary Medicine, Kansas State University, Manhattan, KS 66506, USA; sadetunji@vet.k-state.edu (S.A.A.); ncernic@vet.k-state.edu (N.C.); 2 Center for Grain and Animal Health Research, Agricultural Research Service, United States Department of Agriculture, Manhattan, KS 66502, USA; dmitriy.smolensky@usda.gov; 3 National Bio and Agro-Defense Facility, Agricultural Research Service, United States Department of Agriculture, Manhattan, KS 66502, USA; dana.mitzel@usda.gov (D.N.M.); jeana.owens@usda.gov (J.L.O.); 4 Roman L. Hruska U.S. Meat Animal Research Center, Agricultural Research Service, United States Department of Agriculture, Clay Center, NE 68933, USA; carol.chitkomckown@usda.gov

**Keywords:** arboviruses, cell culture, Japanese encephalitis, infection, in vitro, porcine

## Abstract

Japanese encephalitis virus (JEV) is a zoonotic mosquito-borne pathogen that regularly causes severe neurological disease in humans in Southeast Asia and the Western Pacific region. Pigs are one of the main amplifying hosts of JEV and play a central role in the virus transmission cycle. The objective of this study was to identify in vitro cell systems to investigate early effects of JEV infection including viral replication and host cell death. Here, we demonstrate the susceptibility of several porcine cell lines to the attenuated genotype III JEV strain SA14-14-2. Monolayers of porcine nasal turbinate (PT-K75), kidney (SK-RST), testis (ST), and monocyte-derived macrophage (CΔ2+) cells were infected with SA14-14-2 for up to five days at a multiplicity of infection (MOI) of 0.1. The hamster kidney cell line BHK-21, previously shown to be susceptible to SA14-14-2, was used as a positive control. Culture supernatants and cells were collected between 0 and 120 h post infection (hpi), and monolayers were observed for cytopathic effect (CPE) using brightfield microscopy. The number of infectious virus particles was quantified by plaque assay and cell viability was determined using trypan blue staining. An indirect immunofluorescence assay was used to detect the presence of JEV NS1 antigens in cells infected at 1 MOI. All four porcine cell lines demonstrated susceptibility to SA14-14-2 and produced infectious virus by 12 hpi. Virus titers peaked at 48 hpi in CΔ2+, BHK-21, and SK-RST cells, at 72 hpi in PT-K75, and at 120 hpi in ST cells. CPE was visible in infected CΔ2+ and BHK-21 cells, but not the other three cell lines. The proportion of viable cells, as measured by trypan blue exclusion, declined after 24 hpi in BHK-21 and 48 hpi in CΔ2+ cells, but did not substantially decline in SK-RST, PT-K75 or ST cells. At 48 hpi, JEV NS1 was detected in all infected cell lines by fluorescence microscopy. These findings demonstrate several porcine cell lines which have the potential to serve as useful research tools for investigating JEV infection dynamics and host cell mechanisms in a natural amplifying host species, such as pigs, in vitro.

## 1. Introduction

Arthropod-borne viruses (arboviruses) are common causes of emerging infectious diseases that pose significant public health threats [1,2]. Notable among these viruses is Japanese encephalitis virus (JEV), a zoonotic mosquito-borne flavivirus that is a leading cause of severe neurologic infections in humans in endemic regions [3,4,5]. JEV is highly endemic in the Asia–Pacific region and has expanded geographically to other continents around the world, including Africa and Europe [6,7,8,9,10]. The prevalence of the virus in both temperate and tropical climates, and its demonstrated ability to spread to new geographic regions, represent a significant risk of exposure and infection to people worldwide [11,12,13]. The global incidence of Japanese encephalitis (JE) is estimated to be close to 70,000 cases annually, with up to a 30% case-fatality rate and long-lasting neurological complications in up to 50% of clinical cases, especially in children and immune-compromised adults [3,14,15,16].

JEV is transmitted to humans and other vertebrate hosts through the bites of infected mosquitoes. The main arthropod vector implicated in virus transmission in Southeast Asia is *Culex tritaeniorhynchus* [17,18]. However, more than 30 vector species, including mosquitoes from the *Anopheles*, *Aedes*, *Armigeres,* and *Mansonia* genera, have been identified as competent vectors [19,20,21]. JEV is maintained in a transmission cycle between mosquitoes and vertebrate hosts, including aquatic birds and pigs, thus giving the potential for extensive dissemination and maintenance upon introduction into new areas [10,22]. Humans and horses are considered dead-end hosts of JEV because the virus does not appear to replicate to high enough levels to sustain transmission to competent mosquito vectors and/or susceptible hosts [23]. Pigs are the major amplifying hosts and important reservoirs of JEV mainly because they are highly viremic upon natural infection, facilitating transmission to arthropod vectors [6,15,22]. Under experimental conditions, pigs have also been implicated in vector-free transmission of the virus, suggesting a potential for spread and maintenance of the virus in pig populations even in the absence of competent vectors [22,24,25]. Therefore, pig farming and the presence of feral hogs represent potential risks factors for widespread JEV infection to humans and susceptible domestic animals [6,23,26,27]. The rate and severity of clinical disease in JEV-infected pigs seem to be age-dependent. For example, young piglets are highly susceptible to JEV, presenting with sometimes fatal neurological manifestations whereas adult pigs may present with reproductive complications including abortion and testicular edema [17,28,29,30]. With numerous vectors identified as being competent for JEV, there is a potential for the introduction and spread of JEV to wild and domestic pig populations in new areas, and a risk of economic impacts on pig farming and production [31,32,33,34].

Controlled in vivo challenge models remain the gold standard for investigating the complexities of viral pathogenesis, and recent studies have provided valuable insights toward a better understanding of JEV infections in swine [24,25,27,35,36,37]. However, host and virus factors associated with JEV pathogenesis remain poorly understood and advances in treatment and prevention in swine have been hindered by a lack of thorough understanding of the viral replication cycle and host–virus molecular interactions. In vitro cell model systems can serve as useful tools to investigate these interactions at the mechanistic level. JEV has demonstrated the ability to infect, and replicate in, cell types of multiple origins including immune cells, cells of the central nervous system, epithelial cells, and endothelial cells [38]. The mechanisms of JEV’s entry into host cells are not well understood and may vary by tissue type, with evidence for roles of both receptor-mediated and receptor-independent mechanisms [38,39,40]. Recent in vitro and ex vivo studies with primary porcine cells, including nasal epithelial cells, monocyte-derived macrophages, monocyte-derived dendritic cells, and testicular cells, have provided insights into JEV tropism and innate immune responses [29,41,42]. However, established cell lines are a needed resource because most researchers have limited access or ability to produce primary cell cultures from fresh porcine samples. Few available cell lines have been identified as suitable for the study of JEV. The porcine kidney cell lines PK-15 and PS have been shown to support JEV replication; however, the ability to use them in some regions and/or assays may be impacted by their reported chronic infections with other porcine viruses [43,44]. A commercially available Sertoli cell line derived from porcine testis (ST) was recently shown to be a promising model to study JEV infection and subsequent inflammatory responses in the testis, a known target organ for JEV infection [28,29]. The objective of this study was to identify existing, established porcine cell lines for use as tools to study the pathogenesis of JEV in pigs in vitro, with the hypothesis that they would be susceptible to the attenuated JEV strain, SA14-14-2. We investigated the infection dynamics of SA14-14-2 in commercially available cell lines derived from swine kidneys, nasal turbinates, and testis. Additionally, we examined an established monocyte-derived macrophage cell line, CΔ2+ [45] to determine its potential utility as a model to study JEV infection in innate immune cells which are known targets of JEV infection [42,46].

## 2. Materials and Methods

### 2.1. Virus and Cell Lines

All work was approved by the Kansas State University Institutional Biosafety Committee and conducted in approved biosafety level 2 laboratories at the Center for Grain and Animal Health Research, United States Department of Agriculture (USDA), Agricultural Research Service (ARS), Manhattan, KS, USA.

The JEV SA14-14-2 strain was obtained from the World Reference Center for Emerging Viruses and Arboviruses (WRCEVA) at the University of Texas-Medical Branch, Galveston, TX, USA. Virus was passaged one time in African green monkey Vero cells, then propagated in Baby hamster kidney (BHK-21) cells prior to experiments; titer was determined by standard plaque assay. The Vero, BHK-21, PT-K75 (porcine nasal turbinate), SK-RST (porcine kidney cortex), and ST (porcine testis) cells were obtained from the American Type Culture Collection (ATCC), Manassas, VA, USA. Cell growth media (Thermo Fisher Scientific, Waltham, MA, USA) were as follows: Medium 199, Earle’s Salts (199E; Vero), minimum essential medium (MEM; BHK-21, SK-RST, ST) or Dulbecco’s Modified Eagle Medium (DMEM; PT-K75), supplemented with 10% (v/v) fetal calf serum (FCS, R&D Systems, Minneapolis, MN, USA) and 1× antibiotic-antimycotic (100 units/mL of penicillin, 100 µg/mL of streptomycin, and 0.25 µg/mL Amphotericin B, Thermo Fisher Scientific, Waltham, MA, USA). CΔ2+ cells were initiated and established by the USDA, ARS, U.S. Meat Animal Research Center (USMARC), Clay Center, NE and maintained as previously described [45]. All cells were grown at 37 °C in a 5% CO_2_ atmosphere.

### 2.2. Virus Growth Kinetics and Cell Viability

Cell monolayers of 3 × 10^5^ cells were grown in 6-well plates for approximately 40 h, then infected at 0.1 multiplicity of infection (MOI) with SA14-14-2 in MEM supplemented with 2% FCS and 1× antibiotic-antimycotic (Thermo Fisher Scientific, Waltham, MA, USA) based on cell counts at the time of infection. Virus was adsorbed for 90 minutes, then monolayers were washed twice, and fresh growth media added. Infections were performed three separate times for each cell line. After infection, cell morphology was monitored, and cytopathic effect (CPE) was observed using brightfield microscopy. Cell and supernatant samples were collected at 0, 12, 24, 48, 72, 96, and 120 h post infection (hpi). Cell culture supernatants were harvested to detect extracellular virus released into the culture media, and cell monolayers were trypsinized and collected to detect virus particles that remained cell-associated. Cell viability was determined using a 1:1 dilution with trypan blue (Thermo Fisher Scientific, Waltham, MA, USA), and cells were counted on a Luna-FL cell counter (Logos Biosystems, Annandale, VA, USA) using brightfield settings. All samples were subjected to three freeze/thaw cycles to lyse cells and stored at −80 °C until titered by plaque assay. Standard plaque assays on BHK-21 cells were performed to calculate titers in pfu/mL. To compare the number of viable virions produced in the extracellular and cell-associated fractions of each well, pfu/well was calculated by multiplying the titer of each sample (pfu/mL) by the supernatant volume (3 mL/well), or collection volume of cellular fraction (0.5 mL/well), as applicable.

### 2.3. Immunofluorescence Assay

To demonstrate JEV NS1 protein production, an indirect immunofluorescence assay (IFA) was performed. Briefly, 2–2.5 × 10^4^ cells seeded in black 24-well µ-plates (Ibidi GmbH, Munich, Germany) were infected with JEV at an MOI of 1, or mock infected for 48 h. At 48 hpi, cells were fixed and permeabilized with the Image-iT fixation/permeabilization kit based on the manufacturer’s recommendation (Invitrogen, Carlsbad, CA, USA). To minimize nonspecific background signals, cells were blocked overnight with 3% Bovine Serum Albumin (BSA) at 4 °C. Primary (mouse anti-JEV NS1 clone GT1410—GeneTex, Irvine, CA, USA) and secondary antibodies (Goat anti-mouse IgG highly cross adsorbed, Alexa Fluor 594—Invitrogen, Carlsbad, CA, USA) were added to cell cultures at a dilution of 1:100 for 2 h and 1:500 and incubated for 1 h at room temperature, respectively. Cell nuclei were counterstained with ProLong Diamond Anti-fade Mountant with 4′,6-diamidino-2-phenylindole (DAPI; Invitrogen, Carlsbad, CA, USA) and imaged with a fluorescence microscope (Keyence Corporation, Itasca, IL, USA). To quantify the fluorescence intensity of NS1 antibody signal in JEV-infected cells, the Keyence image analyzer was used to measure the average brightness integration of five 10× objective fields from each of two technical replicates, for each cell line. The percentage of infected cells was calculated as the ratio of the number of NS1-positive cells to the number of DAPI-positive cells, multiplied by 100. The brightness integration and percentage of infected cells were calculated for three independent infection experiments. Graphical representations were performed with GraphPad Prism.

## 3. Results

### 3.1. Porcine Cell Lines Support JEV SA14-14-2 Replication and Produce Infectious Virus

The cell lines selected for this study were chosen based on prior evidence of in vitro or ex vivo JEV susceptibility of related cells or tissues [29,42,45,47,48,49]. When possible, commercial cell lines were chosen owing to their public availability. The selected commercial cell lines had no documented pre-existing infections (natural or transformed), according to the vendor. These cell lines included a cell line with fibroblastic morphology derived from newborn piglet nasal turbinate mucosa (PT-K75), cells with epithelial morphology derived from the kidney cortices of 1-day-old pigs (SK-RST), and ST cells, as discussed above. BHK-21 cells were used as a positive control because they are known to be highly susceptible to JEV infection and have been used extensively in the study of the pathogenetic mechanisms of JEV [48,49]. A non-transformed, commercially available adherent macrophage cell line was not identified; therefore, a previously established macrophage cell line (CΔ2+) derived from the peripheral monocytes of a 10-week-old, mixed-breed pig was used as a model for innate immune cell infection [45]. The attenuated SA14-14-2 vaccine strain of JEV was used for these studies. Wild-type JEV requires containment in a biosafety level (BSL) 3 facility; however, SA14-14-2 is a BSL-2 pathogen that has been a useful surrogate for in-depth study of JEV infection dynamics and host cell mechanisms critical for viral replication [50,51].

**Figure 1:**
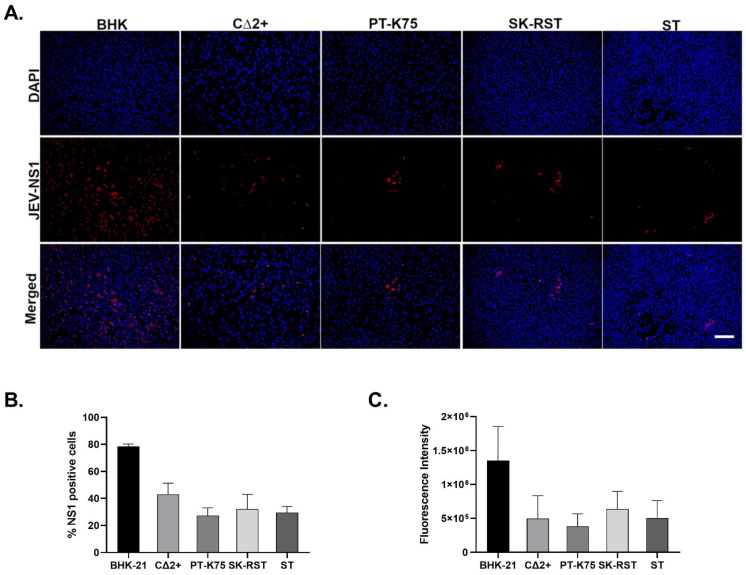
Detection of JEV NS1 in porcine cells infected with JEV SA14-14-2. (**A**) Cells infected with JEV SA14-14-2 at a multiplicity of infection (MOI) of 1 for 48 h were fixed, permeabilized, incubated with a monoclonal antibody for JEV NS1 followed by Alexa Fluor 594 (red) and DAPI (blue), and observed with a Keyence BX-8100 fluorescence microscope. Scale Bar = 100 μm. (**B**) The percentage of infected cells was calculated as the ratio of NS1-positive cells to DAPI-positive cells, multiplied by 100. (**C**) Fluorescence intensity of the NS1 signal was calculated based on mean integrated brightness. B and C were obtained by measuring five 10× objective fields in duplicate from three separate experiments using the Keyence image analyzer. Bars represent arithmetic mean ± SD.

An indirect immunofluorescent assay was used to determine if the selected porcine cell lines infected with SA14-14-2 could support viral protein synthesis. The flavivirus non-structural protein 1 (NS1) is important for virus replication [52,53]; hence, the presence of cell-associated JEV NS1 was examined at 48 hpi following infection at 1 MOI (Figure 1A). NS1 was detected in all JEV-infected cell lines, with the highest proportion of NS1-positive cells among BHK-21 cells (Figure 1B). The fluorescence intensity of NS1 antibody signal was assessed for all infected cell lines using calculated brightness integration (Figure 1C). The highest NS1 fluorescence intensity was observed in BHK cells. Notable variability was observed in the staining intensity among CΔ2+ cells (Figure 1A). This may be a reflection of heterogeneity within the population, which is consistent with previous observations that cell surface expression of porcine cluster of differentiation (CD) markers can vary between cells [45]. No NS1-specific signal was detected in uninfected controls (Figure S1).

**Figure 2:**
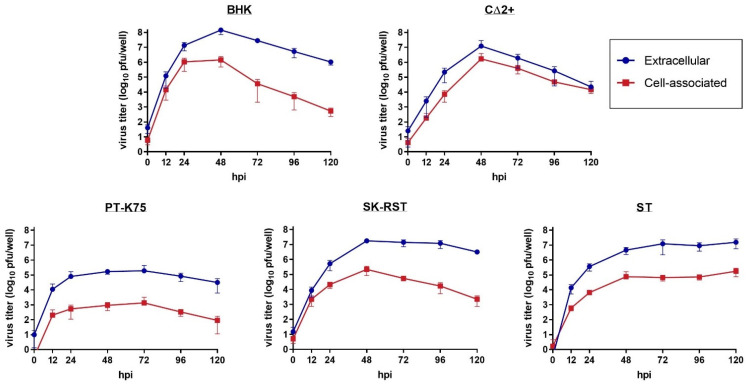
Replication of JEV SA14-14-2 in porcine cell lines. Cell monolayers were infected with JEV SA14-14-2 at a multiplicity of infection (MOI) of 0.1; cells and supernatants were collected at 0, 12, 24, 48, 72, 96, and 120 hpi. Viral titers were quantified by standard plaque assay and expressed as pfu/well for cell-associated (red) and extracellular (blue) fractions. Error bars represent arithmetic mean ± SD of three biological replicates.

To examine the permissiveness of CΔ2+, PT-K75, SK-RST, and ST cells to virus replication and production of infectious virions, JEV-infected cells and corresponding media supernatants were collected at 0, 12, 24, 48, 72, 96, and 120 hpi and virus replication kinetics were analyzed by plaque assay for both fractions at each timepoint (Figure 2). All four porcine cell lines demonstrated susceptibility to SA14-14-2 and produced detectable virus titers by 12 hpi. The extracellular and cell-associated virus titers peaked at the same timepoint for each cell line; BHK-21, CΔ2+, and SK-RST cultures peaked at 48 hpi, while PT-K75 titers peaked at 72 hpi. Virus titers in ST cultures continued to increase through 120 hpi, therefore it is not known if maximum virus production for this cell line was reached by the experimental endpoint. The highest virus titers for each cell line during the five-day study were in the supernatant fractions as provided in Table 1. After peak virus production, the virus titers of the BHK-21 and CΔ2+ cell and supernatant fractions declined rapidly. Extracellular titers of SK-RST and PT-K75 cultures plateaued followed by a moderate decrease, with a more rapid decrease in the cell-associated titers. For all cell lines apart from CΔ2+, the cell-associated virus titers were approximately 2 log_10_ pfu lower than those in the respective extracellular fractions (range 81–141-fold). By comparison, cell-associated virus titers produced by CΔ2+ cells at the 48 hpi peak were 7-fold lower than extracellular titers, and virus production detected in cells and supernatants of that cell line were largely similar throughout the study.

### 3.2. JEV SA14-14-2 Induces Differential Cytopathic Changes in Porcine Cell Lines

**Figure 3:**
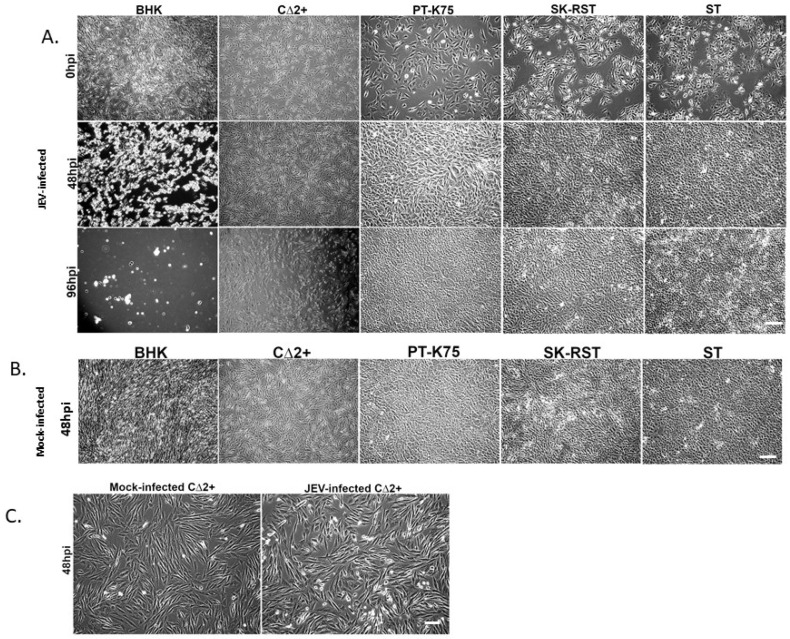
Cytopathic changes observed in porcine cell lines at 0, 48, and 96 hpi. Cells were (**A**) infected with JEV SA14-14-2 or (**B**) mock infected (48 hpi) at a multiplicity of infection (MOI) of 0.1. Morphological changes were observed using brightfield microcopy; Scale Bar = 300 μm. (**C**) Mock-infected and infected CΔ2+ cells at 48 hpi shown at higher magnification; Scale Bar = 100 μm.

**Figure 4:**
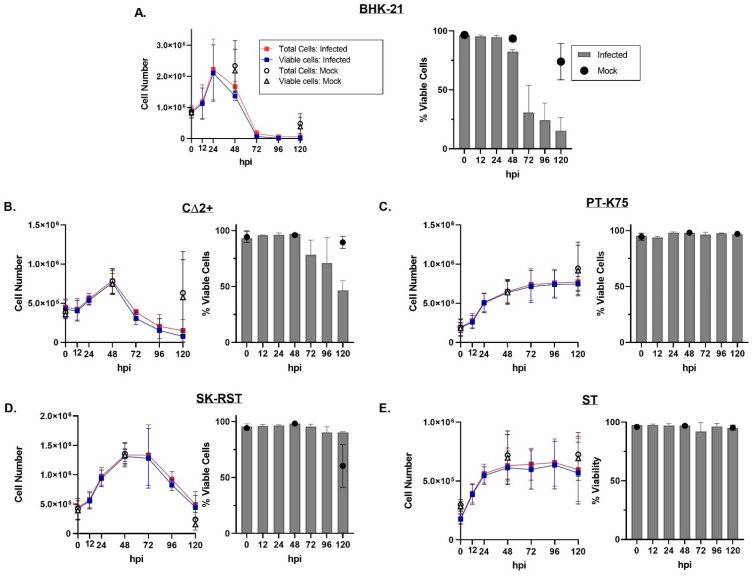
Porcine cell numbers and viability following infection with JEV SA14-14-2. BHK-21 (**A**), CΔ2+ (**B**), PT-K75 (**C**), SK-RST (**D**), and ST (**E**) cells were infected with JEV SA14-14-2 at a multiplicity of infection (MOI) of 0.1 and collected at 0, 12, 24, 48, 72, 96, and 120 hpi; mock-infected samples were collected at 0, 48, and 120 hpi. Left panels show the absolute numbers of total cells and viable cells per well at each timepoint based on trypan blue exclusion. Right panels show the percent viability at each timepoint. Bars represent arithmetic mean ± SD of three biological replicates.

Cell monolayers were infected with SA14-14-2 at an MOI of 0.1 and evaluated for CPE for 5 days (Figure 3 and Figure S2). BHK-21 cells, which are known to exhibit severe CPE and produce virus at high titers following JEV infection [47,49,54,55] showed marked time-dependent CPE with profound cytolysis evident by 48 hpi (Figure 3A,B). Morphological changes in JEV-infected CΔ2+ cells were first observed at 48 hpi with some increased cell rounding and refractility (Figure 3C). CPE progressed in a time-dependent fashion with CΔ2+ cell detachment evident beginning at 72 hpi, and more than half of the cells appearing detached by 96 hpi (Figure S2). Infection-specific morphologic changes were not evident in PT-K75, SK-RST, and ST cells throughout the infection period. At 120 hpi, the mock-infected BHK-21 and SK-RST cultures showed evidence of substantial overgrowth with sloughing and piling, respectively, of the cell monolayers. The number of cells collected and the proportion of viable infected cells, as measured by trypan blue exclusion, was largely consistent with the cytolysis observed microscopically at early timepoints (Figure 4 and Figure S2), with cell death in infected BHK-21 and CΔ2+ corresponding to loss of monolayer integrity at 48 and 72 hpi, respectively. After an initial proliferation phase, the numbers and viability of infected cells declined after 24 hpi in BHK-21 (Figure 4A) and 48 hpi in CΔ2+ cultures (Figure 4B) but did not substantially decrease in PT-K75 or ST wells (Figure 4C,E). The numbers of infected SK-RST cells (Figure 4D) appeared to decline sharply after 72 hpi; however, dissociation-resistant cell clumping was prevalent in both the mock-infected and infected samples from this cell line at 96 and 120 hpi which likely reduced cell counting accuracy, as supported by the discrepancy between cell monolayer confluency (Figure S2) and cell counts of the later timepoints. Some cell clumping was also observed in ST cells collected at 96 and 120 h.

## 4. Discussion

Pigs have an important role in JE epidemiology as amplifying hosts; however, significant knowledge gaps remain about JEV pathogenesis in these animals. Understanding fundamental mechanisms in the progression of porcine infections is complicated by factors including variable clinical presentations, limited availability of BSL-3 facilities to study wild-type JEV, and the expertise and resources needed to conduct in vivo experiments. To help mitigate some of these obstacles faced by researchers, we aimed to identify in vitro cell systems to facilitate the study of JEV pathogenesis and host–virus interactions in pigs at the cellular level. We demonstrated the susceptibility of several established porcine cell lines, including monocyte-derived macrophages (CΔ2+), nasal turbinate (PT-K75), kidney (SK-RST), and testis (ST) cells, to the attenuated strain of JEV SA14-14-2. This strain, which was derived from the virulent parental strain JEV SA14 through several passages in cultured cells (primary hamster kidney and chicken embryo cells) and tissues of animals (mice and hamsters), has been used for more than two decades as a vaccine to protect against JE in endemic countries [14,15,22,50,56]. SA14-14-2 has previously been described to differ from its parental strain by 57 nucleotide changes and its attenuation enables it to be used at the BSL-2 level, making it more available to researchers than wild-type strains [57]. Despite its attenuation, studies have shown that viral replication and protein expression profiles of SA14-14-2 are as efficient as that of its parent strain with slight differences in growth kinetics, plaque sizes, and time in which maximum titer was achieved [50,57], and it has been used in some in vivo animal models [50,51,57]. It remains to be investigated how observations made here with SA14-14-2 will translate to in vivo pathogenesis in pigs infected with virulent strains of JEV or how the findings with this genotype III virus will extrapolate to pathogenesis associated with other genotypes in vivo or in vitro. However, despite these potential limitations, the in vitro system described here can be a useful surrogate to study pig–virus interactions and perform discovery of disease countermeasures such as antiviral compounds at lower levels of biocontainment.

Macrophages represent an important cell population that warrants in-depth evaluation in order to understand the pathogenesis of JEV infection. Host defenses against JEV are facilitated by innate immune cells including macrophages [46]; however, tissue macrophages and microglia of the central nervous system can also contribute to the severity of JE through the induction of an inflammatory cytokine milieu [58,59]. Studies have shown that human JE patients and mouse models of JEV infection demonstrate increased circulation of monocytes in the peripheral blood and infiltration of macrophages into the CNS [58,60]. Generally, primary pig macrophages are labor intensive to isolate and vary in quantity, quality, and activity; hence, we used the cell line CΔ2+, which has been characterized as a representative macrophage line based on cell morphology, proinflammatory cytokine expression, cell-surface markers, and bactericidal activities [45]. In agreement with previous studies showing JEV infection of monocyte-derived macrophages of pigs, humans, and mice, CΔ2+ cells demonstrated remarkable susceptibility to JEV, successfully supporting replication of the virus and producing high titers of infectious virus particles [41,61]. Of the four porcine cell lines, CΔ2+ cells exhibited the most cytopathology with some observable cell rounding and decreased viability after 48 hpi, which coincided with peak virus production in both the cell-associated and extracellular compartments. In contrast to the other three porcine cell lines, the quantity and kinetics of infectious virus production in the supernatants were similar to the cell-associated titers throughout the five-day infection period. The significance of this is not clear but could indicate functional bottlenecks in virus release or potential cellular mechanisms favoring intracellular survival and persistence. Macrophages have been implicated in the persistence and pathogenesis of varying pig infectious agents [8,62,63,64]. The demonstrated permissiveness of CΔ2+ cells indicate that this cell line is a useful tool to better understand viral replication mechanisms and host defense factors against JEV infection in the innate immune cells of a natural animal host.

Like CΔ2+ cells, SK-RST and ST cells produced peak infectious virus titers that were within approximately 1 log_10_ pfu/mL of titers produced by highly permissive BHK-21 cells (range 0.8–1.3). Unlike CΔ2+ cells, however, SK-RST and ST cells did not exhibit obvious CPE during the study. This lack of cytolysis may make these cells unsuitable reporter cells for assays such as plaque assays. However, they may be useful cells for virus propagation due to the ability to maintain intact monolayers while producing high virus titers. Although cell clumping reduced the ability to accurately quantify the numbers of SK-RST and ST cells that remained during the last two timepoints of infection, the proportion of viable cells did not substantially decline throughout the infection period, suggesting that JEV had less of an effect on cell survival mechanisms in these cells than on BHK-21 and CΔ2+. The effects of JEV on the viability and death of these cells will be further explored with additional assays in future studies.

The high virus titers produced by ST cells in this study also provide insights into the utility of studying SA14-14-2 in porcine cell lines to investigate JEV pathogenesis in pigs at the BSL-2 level. JEV is known to cause testicular edema in boars and studies investigating the impact of JEV on the male reproductive system are limited [29]. ST cells have previously been shown to be highly permissive for wild-type JEV infection [29]. Here, we demonstrate that ST cells are likewise highly permissive for the attenuated vaccine strain SA14-14-2.

Data from the current study demonstrated that the porcine nasal turbinate cell line PT-K75 produced infectious JEV with peak titers 2–2.5 log_10_ pfu/mL lower than the other porcine cell lines. The comparison of extracellular virus to cell-associated virus indicates that virus was not being trapped inside the cells, so it is possible that this cell line is less permissive for JEV, or the attenuation of SA14-14-2 may be contributing to the lower titers. Notwithstanding, PT-K75 cells may serve as an in vitro alternative to in vivo or ex vivo models for some studies of vector-free transmission of JEV. Studies have shown the ability of the virus to propagate in the nasal mucosa, one of the targets of virus infection during experimentally demonstrated vector-independent transmission between pigs, indicating a potential means of virus spread and maintenance in pig populations [25,41]. Hence, these cells can be further used to study the replication mechanisms in the targets of oronasal route of virus transmission. Planned studies include investigating the behavior of wild-type strains of JEV in PT-K75, as well as the other porcine cell lines.

Overall, results from the current study demonstrated that JEV induced productive infections in several established pig cell lines, most of which are commercially available. These cell lines represent research resources that can assist in the study of JEV biology and the evaluation of host factors and disease mechanisms in a natural amplifying host. Additionally, they may be useful tools for countermeasure discovery such as therapeutic target screening and vaccine development, with the ultimate goals of improving treatments and preventing disease in humans and animals.

## Figures and Tables

**Table 1:** Peak virus titers of each cell line measured during study.

Cell Line	Peak Titer ± SD (pfu/mL)	Time of Peak Titer (hpi)
BHK-21	4.83 ± 2.4 × 10^7^	48
C∆2+	4.11 ± 5.54 × 10^6^	48
SK-RST	5.83 ± 1.44 × 10^6^	48
PT-K75	6.38 ± 8.08 × 10^4^	72
ST	5.22 ± 3.22 × 10^6^	120 *

Peak titers are expressed as the arithmetic mean of three biological replicates. * Titers were still rising at the last timepoint (120 hpi); therefore, it is not known whether the peak titer represents maximum virus production for this cell line.

## Data Availability

The data presented in this study are available on request from the corresponding author.

## Supplementary Materials

The following are available online at https://www.mdpi.com/article/10.3390/pathogens10111468/s1, Figure S1. Comparison of JEV NS1 in infected and uninfected porcine cells. Figure S2. Cytopathic changes observed in porcine cell lines.
