# Supplementary material for: In Vitro Infection Dynamics of Japanese Encephalitis Virus in Established Porcine Cell Lines
Source: Pathogens. 2021 Nov 12;10(11):1468. doi: 10.3390/pathogens10111468 (PMC8618157; doi:10.3390/pathogens10111468)
Supplement: Supplementary file 1 [file pathogens-10-01468-s001.zip › pathogens-1301561-supplementary.pdf]

S1

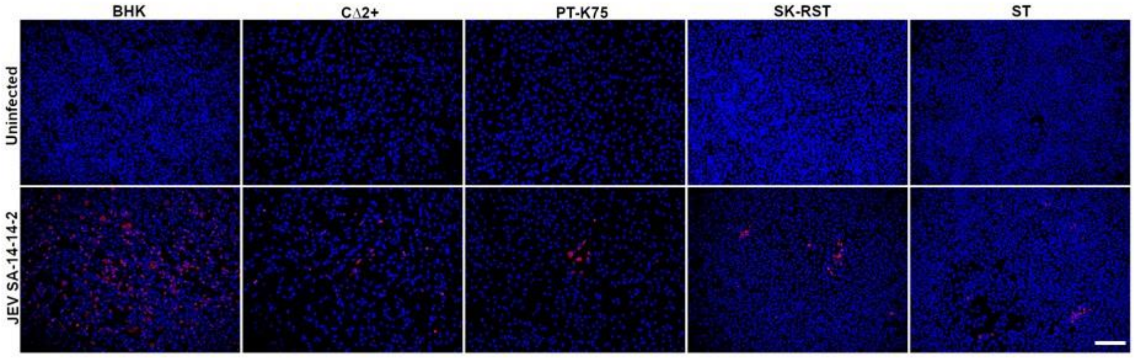

Supplementary Figure S1. Comparison of JEV NS1 in infected and uninfected porcine cells. Cells were mock infected (top row) or infected with JEV SA14-14-2 at a multiplicity of infection (MOI) of 1 (bottom row). At 48 hpi cells were fixed, permeabilized, incubated with a monoclonal antibody for JEV NS1 followed by Alexa Fluor 594 (red) and DAPI (blue), and observed with a Keyence BX-8100 fluorescence microscope. Scale Bar = 100μm.

S2

A.

JEV-infected

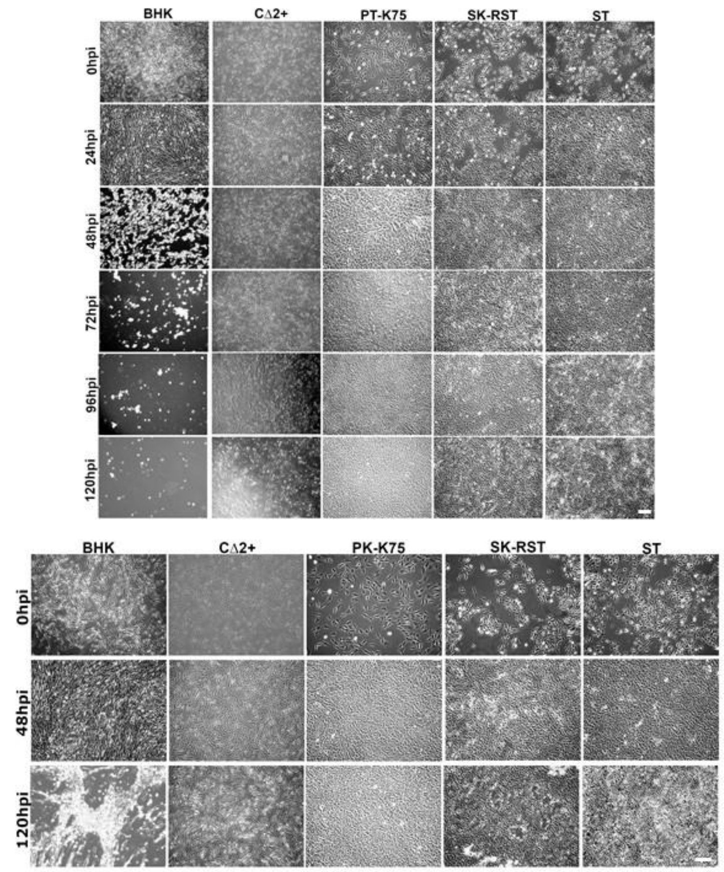

B.

Mock-infected

Supplementary Figure S2. Cytopathic changes observed in porcine cell lines. (A) Cells infected with JEV SA14-14-2 at a multiplicity of infection (MOI) 0.1 were observed for morphological changes at 0, 24, 48, 72, 96, and 120 hpi using brightfield microcopy. (B) Mock-infected cells were photographed at 0, 48, and 120 hours. Scale Bar = 300μm.
